# Draft Genome Sequence of *Bacillus cereus* Strain BcFL2013, a Clinical Isolate Similar to G9241

**DOI:** 10.1128/genomeA.00469-14

**Published:** 2014-05-29

**Authors:** Jay E. Gee, Chung K. Marston, Scott A. Sammons, Mark A. Burroughs, Alex R. Hoffmaster

**Affiliations:** a Bacterial Special Pathogens Branch, Division of High-Consequence Pathogens and Pathology, National Center for Emerging and Zoonotic Infectious Diseases, Centers for Disease Control and Prevention, Department of Health and Human Services, Atlanta, Georgia, USA; b Biotechnology Core Facility Branch, Division of Scientific Resources, National Center for Emerging and Zoonotic Infectious Diseases, Centers for Disease Control and Prevention, Department of Health and Human Services, Atlanta, Georgia, USA

## Abstract

*Bacillus cereus* strains, such as G9241, causing anthrax-like illnesses have recently been discovered. We report the genome sequence of a clinical strain, *B. cereus* BcFL2013, which is similar to G9241, recovered from a patient in Florida.

## GENOME ANNOUNCEMENT

*Bacillus cereus* is generally a harmless bacterium and is common in the environment. Strains of this species that cause disease are typically associated with food-borne emesis and diarrhea. More rarely is it associated with serious illness. *B. cereus* strains with similarities to *Bacillus anthracis*, the cause of anthrax, have been identified recently, which are linked to serious cases of pneumonia (1–3). The first *B. cereus* strain characterized, G9241, was isolated from a case of pneumonia in a welder in Louisiana in 1994 (2). In 2003, three more strains from Texas were isolated, including two clinical isolates, *B. cereus* 03BB87 and 03BB102, associated with two fatal cases of pneumonia (1). The features of interest in G9241 associated with virulence are the presence of two plasmids, pBCXO1 and pBC210. pBCXO1, homologous to plasmid pXO1 of *B. anthracis*, contains the genes encoding anthrax toxins (*pagA, cya*, and *lef*), as well as *hasACB*, which encodes hyaluronic acid capsule formation. pBC210 contains *bpsXABCDEFGH*, which encodes an exopolysaccharide (4). In 2013, we investigated a case of human cutaneous infection in Florida, which was similar in appearance to an anthrax eschar. The investigation yielded isolate *B. cereus* BcFL2013, which has features similar to those of G9241.

Here, we report the draft genome sequence of strain BcFL2013. A 101 × 101 paired-end run was performed on an Illumina GAIIx using TruSeq chemistry and yielded 10,182,400 reads. A *de novo* assembly was performed using CLC Genomics Workbench 6.0.4 (CLC Inc., Aarhus, Denmark) and yielded 84 contigs consisting of 5.46 Mb, with an *N*_50_ of 139,961 bp.

We analyzed the genome of BcFL2013 *in silico* by multilocus sequence typing (MLST) which uses the sequences of seven housekeeping genes to assign sequence types (ST) (5). We found that BcFL2013 belongs to ST78, matching G9241 and 03BB87. We determined that it has one of the operons associated with capsule production in G9241, *hasACB*, also associated with pBCXO1, which consists of the hyaluronan synthase (*hasA*), UTP-glucose-1-phosphate uridylyltransferase (*hasC*), and UDP-glucose 6-dehydrogenase (*hasB*) genes, with each gene having 100% identity with the corresponding gene from G9241. We did not detect the other G9241 capsule operon, *bpsXABCDEFGH*. We mapped the genome of BcFL2013 to that of plasmid pBCXO1 of G9241, which indicated that a homolog of this plasmid is present with >99.98% identity, but it included a 2.5-kb deletion. We determined that the anthrax toxin genes *pagA*, *cya*, and *lef* were also present and that the sequences have 100% identity to those found in G9241. Mapping the genome of BcFL2013 to plasmid pBC210 of G9241 indicated a homolog of the plasmid was present but had only approximately half the sequence of the 209,385 bp noted for pBC210. The study of the genome sequences of these *B. cereus* strains with characteristics more often associated with *B. anthracis* will provide insights into their potential for virulence.

### Nucleotide sequence accession numbers.

This whole-genome shotgun project has been deposited at DDBJ/EMBL/GenBank under the accession no. JHQN00000000. The version described in this paper is version JHQN01000000.
